# Comparison of Heuristic Algorithms in Identification of Parameters of Anomalous Diffusion Model Based on Measurements from Sensors

**DOI:** 10.3390/s23031722

**Published:** 2023-02-03

**Authors:** Rafał Brociek, Agata Wajda, Damian Słota

**Affiliations:** 1 Department of Mathematics Applications and Methods for Artificial Intelligence, Faculty of Applied Mathematics, Silesian University of Technology, 44-100 Gliwice, Poland; 2 Institute of Energy and Fuel Processing Technology, 41-803 Zabrze, Poland

**Keywords:** inverse problem, minimum of function, heuristic algorithm, fractional differential equation, parameter identification

## Abstract

In recent times, fractional calculus has gained popularity in various types of engineering applications. Very often, the mathematical model describing a given phenomenon consists of a differential equation with a fractional derivative. As numerous studies present, the use of the fractional derivative instead of the classical derivative allows for more accurate modeling of some processes. A numerical solution of anomalous heat conduction equation with Riemann-Liouville fractional derivative over space is presented in this paper. First, a differential scheme is provided to solve the direct problem. Then, the inverse problem is considered, which consists in identifying model parameters such as: thermal conductivity, order of derivative and heat transfer. Data on the basis of which the inverse problem is solved are the temperature values on the right boundary of the considered space. To solve the problem a functional describing the error of the solution is created. By determining the minimum of this functional, unknown parameters of the model are identified. In order to find a solution, selected heuristic algorithms are presented and compared. The following meta-heuristic algorithms are described and used in the paper: Ant Colony Optimization (ACO) for continous function, Butterfly Optimization Algorithm (BOA), Dynamic Butterfly Optimization Algorithm (DBOA) and Aquila Optimize (AO). The accuracy of the presented algorithms is illustrated by examples.

## 1. Introduction

With the increase in computing power of computers, all kinds of simulations of various phenomena occurring, among others, in physics, biology and technology are gaining in importance. The considered mathematical models are more and more complicated and can be used to model various processes in nature, science and engineering. In the case of modeling anomalous diffusion processes (e.g., heat conduction in porous materials) or processes with long memory, fractional derivatives play a special role. There are many different fractional derivatives, in which the following are the most popular: Caputo, Riemann-Liouville and Riesz. Authors of the study [1] present a model dedicated risk of corporate default, which can be described as a fractional self-exciting model. The model and methods introduced in the study were used to carry out a validation on real market data. In result, the fractional derivative model became better. Ming et al. [2] used Caputo fractional derivative to simulate China’s gross domestic product. The fractional model was compared with the model based on the classical derivative. Using the fractional derivative, the authors built a better and more precise model to predict the values of gross domestic product in China. Another applications of fractional derivatives in modeling processes in biology can be found in the article [3]. The authors presented the applications of the Atangan-Baleanu fractional derivative to create models of such processes as: Newton’s law of cooling, population growth model and blood alcohol model. In the article [4], authors used Caputo fractional derivative to investigate and model population dynamics among tumor cells-macrophage. The study also estimated unknown model parameters based on samples which were collected from the patient with non-small cell lung cancer who had chemotherapy-naive hospitalized. De Gaetano et al. [5] presented a mathematical model with a fractional derivative for Continuous Glucose Monitoring. The paper also contains the numerical solution of the considered fractional model. Based on experimental data from diabetic patients, the authors determine the order of the fractional derivative for which the model best fits the data. The research shows that the fractional derivative model fits the data better than the integer derivative model (both first and second order). More about fractional calculus and its application can be found in [6,7,8].

In order to implement more and more accurate and faster computer simulations, it is necessary to improve various types of numerical methods or algorithms that solve direct and inverse problems. Solving the inverse problem allows to design the process and select the input parameters of the model in a way that make possible obtaining the desired output state. Such tasks are considered difficult due to the fact that they are ill conditioned [9]. Sensor measurements often provide additional information for inverse issues. Based on these measurements, the input parameters of the model are selected and the entire process is designed. In the study [10] a variational approach for reconstructing the thermal conductivity coefficient is presented. The authors also cite statements regarding the existence and uniqueness of the solution. Numerical examples are also provided. In the article [11] the solution of the inverse problem consists in identifying the coefficients of the heat conduction model based on temperature measurements from sensors. In addition, several mathematical models were compared, in particular fractional models with classical model. Under the study, the parameters like order of fractional derivative as well as thermal conductivity and heat transfer coefficient were identified. Considerations regarding solving the inverse problem are also included in the article [12]. The authors present the approach of the solution from the Deep Neural Network, in which they used deep-learning methods. It allowed for learning all free parameters and functions through training. The back-propagation of the training data can be one of the methods for training the deep network. More examples of inverse problems in mathematical modeling and simulations can be found in [13,14,15,16,17,18,19,20].

In this article, the mathematical model of heat conduction with Riemann-Liouville fractional derivative is presented. In the provided model, the boundary conditions of the second and third order are adopted. Then, a solution of direct problem is shortly described. To solve this problem a finite difference scheme is derived. The inverse problem posed in this article consists in the reconstruction of the third order boundary condition and the identification of such parameters as order of fractional derivative and thermal conductivity. In the process of developing a procedure that solves the inverse problem, a fitness function is created. It describes the error of the approximate solution. In order to identify the parameters, the minimum of this function should be found. The following algorithms are used and compared to minimize the fitness function: Ant Colony Optimization (ACO), Dynamic Butterfly Optimization Algorithm (DBOA) and Aquila Optimization (AO). The presented procedure has been tested on numerical examples.

## 2. Anomalous Diffusion Model

We consider an anomalous diffusion equation in the form of a differential equation with a fractional derivative with over spatial variable:

(1) $$c\;\varrho \;\frac{\partial T(x,t)}{\partial t}=\hat{\lambda }\;\frac{\partial^{\beta }T\left(x,t\right)}{\partial x^{\beta }},\;\;x\in \left(x_{L},x_{R}\right),\;\;t\in \left(0,t_{end}\right).$$

In this approach, the considered anomalous diffusion equation describes the phenomenon of heat flow in porous medium [11,21,22]. In Equation (1) we assume the following notations: T[K]—temperature, x[m]—spatial variable, t[s]—time, $c\;\left[\frac{J}{kg\;K}\right]$—specific heat, $\varrho \;\left[\frac{kg}{m^{3}}\right]$—density, β∈(1,2)—order of derivative and $\hat{\lambda }=\hat{w}\lambda \;\left[\frac{W}{m^{3-\beta }K}\right]$ is scaled heat conduction coefficient, where $\hat{w}$ is scale parameter. Heat conduction λ had to be scaled to keep the units consistent. To Equation (1) an initial condition is added:

(2) $$T\left(x,0\right)=\psi \left(x\right),\;\;\;x\in \left[x_{L},x_{R}\right].$$

On the left side of the spatial interval the homogeneous boundary condition of the second order is taken:

(3) $$-\lambda \frac{\partial T(x,t)}{\partial x}{|}_{x=x_{L}}=0,\;\;\;t\in \left(0,t_{end}\right],$$

and for the right boundary of the spatial interval the boundary condition of the third order is assumed:

(4) $$-\lambda \frac{\partial T(x,t)}{\partial x}{|}_{x=x_{R}}=h\left(t\right)\left(T\left(x_{R},t\right)-T_{\infty }\right),\;\;\;t\in \left(0,t_{end}\right].$$

The symbols $T_{\infty }$, *h* appearing in the Equation (4) denote ambient temperature and the heat transfer coefficient.

In the Equation (1) there is a fractional derivative with respect to space, which is defined as the Riemann-Liouville derivative [23]:

(5) $$\frac{\partial^{\beta }T\left(x,t\right)}{\partial x^{\beta }}=\frac{1}{\Gamma (2-\beta )}\frac{\partial^{2}}{\partial x^{2}}\int_{x_{L}}^{x}T\left(s,t\right){\left(x-s\right)}^{1-\beta }\;ds,\;\beta \in \left(1,2\right).$$

## 3. Numerical Solution of Direct Problem

In order to solve the direct problem for model (1)–(4) it is used finite difference scheme. The considered area is discretized by creating a mesh $S=\{\left(x_{i},t^{k}\right):x_{i}=x_{L}+i\;\Delta x$, $t^{k}=k\;\Delta t\}$, where $\Delta x=(x_{R}-x_{L})/M$ and $\Delta t=t_{end}/K$ and i=0,…,M,k=0,…,K. Then the Riemann-Liouville derivative has to be approximated [23]:

(6) $$\frac{\partial^{\beta }T\left(x_{i},t^{k}\right)}{\partial x^{\beta }}\approx \sum_{j=0}^{i+1}\frac{\Gamma (j-\beta )}{\Gamma (-\beta )\Gamma (j+1)}T_{i-j+1}^{k},$$

as well as boundary conditions (3) and (4):

(7) $$-\lambda_{0}\left(\frac{-T_{2}^{k+1}+4T_{1}^{k+1}-3T_{0}^{k+1}}{2\Delta x}\right)=q^{k+1},$$

(8) $$-\lambda_{N}\left(\frac{T_{M-2}^{k+1}-4T_{M-1}^{k+1}+3T_{M}^{k+1}}{2\Delta x}\right)=h^{k+1}\left(T_{M}^{k+1}-T^{\infty }\right),$$

where $T^{\infty }$ is the ambient temperature, $T_{i}^{k}$ is the approximate value of the function *T* in point $\left(x_{i},t^{k}\right)$, and *h* is a function describing the heat transfer coefficient. Using the Equations (6)–(8) we obtain a differential scheme (a system of equations). By solving this system, the values of the function *T* will be determined in mesh points.

## 4. Inverse Problem and the Procedure for Its Solution

The problem considered in this article concern the inverse problem. It consists in establishing the input parameters of the model in a way that allows obtaining the temperature at the boundary corresponding to the measurements from the sensors. The identified parameters are: thermal conductivity $\hat{\lambda }$, order of derivative β and heat transfer function *h* in the form of a second degree polynomial. In the presented approach, after solving the direct problem for fixed values of unknown parameters, we obtain approximation of *T* and compare it to the measurements data. This is a method of creation the fitness function:

(9) $$F\left(\hat{\lambda },\beta ,h\right)=\sum_{j=1}^{N}{\left(T_{j}\left(\hat{\lambda },\beta ,h\right)-T_{j}^{m}\right)}^{2},$$

where *N* is a number of measurements, $T_{j}\left(\hat{\lambda },\beta ,h\right)$ are temperature values at the measurement point calculated from the model, and $T_{j}^{m}$ are measurements from sensors. To find the minimum of function (9) we use selected metaheuristic algorithms described in Section 5.

## 5. Meta-Heuristic Algorithms

In this section, we present selected metaheuristic algorithms for finding the minimum of functions. These algorithms will be: Ant Colony Optimization (ACO) for continuous function optimization, Dynamic Butterfly Optimization Algorithm (DBOA) and Aquila Optimization (AO).

### 5.1. ACO for Continuous Function Optimization

The inspiration for the creation this minimum function search algorithm was the observation of the habits of ants while searching for food. In the first stage, the ants randomly search the area around their nest. In the process of foraging for food, ants secrete a chemical called a pheromone. Thanks to this substance, the ants have a chance to communicate with each other. The amount of secreted substance depends on the amount of food found. If the ant has successfully found a food source, the next step is to return to the nest with a food sample. The animal leaves a pheromone trail that will allow other ants to find the food source. This mechanism was adapted to create the ACO algorithm for continuous function optimization [24]. More on the algorithm and its applications can be found, among others, in articles [25,26,27,28].

There are three main parts to the algorithm:

- Solution (pheromone) representation. Points from the search area $R^{n}$ are identified as pheromone patches. In other words, the pheromone spot plays the role of a solution. Thus, *k*-th pheromone spot (or approximate solution) can be represented as $x^{k}=\left(x_{1}^{k},x_{2}^{k},\ldots ,x_{n}^{k}\right)$. Each solution (pheromone spot) has its quality calculated on the basis of fitness function $F(x^{k})$. In each iteration of the algorithm, we store a fixed number of pheromone spots in the set of solutions (establish at the start of the algorithm).
- Transformation of the solution by the ant. The procedure of constructing a new solution, in the first place, consists in choosing one of the current solutions (pheromone spots) with a certain probability. The quality of the solution is a factor that determines the probability. The relationship here is as follows: with the increase in the quality of the solution, the probability of selection increases. In this paper, the following formula is adopted to calculate the probability (based on the rank) of the *k*-th solution:
  (10) $$p_{k}=\frac{\omega_{k}}{\sum_{j=1}^{L}\omega_{j}},$$
  where *L* denotes number of all pheromone spots, and ω is expressed by the formula:
  (11) $$\omega_{k}=\frac{1}{qL\sqrt{2\pi }}e^{-\frac{{\left(rank\left(k\right)-1\right)}^{2}}{2{\left(qL\right)}^{2}}}.$$
  The symbol rank(k) in the Equation (11) denotes the rank of the *k*-th solution in the set of solutions. The parameter *q* is a parameter that narrows the search area. In case of small value of *q*, the choice of the best solution is preferred. The greater *q*, the closer the probabilities of choosing each of the solutions. After choosing *k*-th solution, it is required to perform Gaussian sampling using the formula:
  (12) $$g\left(x,\mu ,\sigma \right)=\frac{1}{\sigma \sqrt{2\pi }e^{-\frac{{\left(x-\mu \right)}^{2}}{2\sigma^{2}}}},$$
  where $\mu =x_{i}^{k}$ is *i*-th coordinate of *k*-th solution and $\sigma =\frac{\xi }{L-1}\sum_{j=1}^{L}\left|x_{i}^{j}-x_{i}^{k}\right|$ is the calculated average distance between the chosen *k*-th solution and all the other solutions.
- Pheromone spots update. In each iteration of the ACO algorithm, *M* of new solutions is created (*M* denotes the number of ants). These solutions should be included in the solution set. In total, there are L+M of pheromone spots in the set. Then the spots (solutions) are sorted by quality. The worst solutions in the *M* set are removed. Thus, the solution set always has a fixed number of elements equal to *L*.

Pseudocode ACO algorithm for continuous function optimization is presented in Algorithm 1.
**Algorithm 1** Pseudocode of ACO algorithm.1:                **Initialization part.**2: Configuration of ACO algorithm parameters.3: Initialization of starting population $\left\{x^{1},x^{2},\ldots ,x^{L}\right\}$ in a random way.4: Calculation value of the fitness function *F* for all pheromone spots and sorting them according to their rank (quality).5:                 **Iterative part.**6: **for**
iterationi=1,2,…,I
**do**7:  Assignment of probability to pheromone spots according to the Equation (10).8:  **for** antm=1,2,…,M **do**9:    The ant chooses the *k*-th (k=1,2,…,L) solution with probability $p_{k}$.10:    **for** coordinatej=1,2,…,n **do**11:      Using the probability density function (12) in the sampling process, the ant changes the *j*-th coordinate of the *k*-th solution.12:    **end for**13:  **end for**14:  Calculation the value of the fitness function *F* for *M* new solutions.15:  Adding *M* new solutions to the set of archive of old, sorting the archive by quality and then rejection of the *M* worst solutions.16: **end for**17: **return** best solution $x_{best}$.


### 5.2. Dynamic Butterfly Optimization Algorithm

Another of the presented heuristic algorithms is an improved version of the Butterfly Optimization Algorithm (BOA), namely the Dynamic Butterfly Optimization Algorithm (DBOA) [29].

In order to communicate, search for food, connect with a partner, and to escape from a predator, these animals use the sense of smell, taste and touch. The most important of these senses is smell. Thanks to the sense of smell butterflies look for food sources. Sensory receptors, called chemoreceptors, are scattered all over the body of a butterfly (e.g., on the legs).

Scientists studying the life of butterflies have noticed that these animals locate the source of a fragrance with great precision. In addition, they can distinguish fragrances and recognize their intensity. Those were an inspiration for the development of the Butterfly Optimization Algorithm (BOA) [30]. Each butterfly emits a specific fragrance of a given intensity. Spraying the fragrance allows other butterflies to recognize it and then communicate with each other. In this way, a “collective knowledge network” is created. The global optimum search algorithm is based on the ability of butterflies to sense the fragrance. If the animal cannot sense the fragrance of the environment, its movement will be random.

The key concept is fragrance and the way it is received and processed. The concept of modality detection and processing (fragrance) is based on the following parameters: stimulus intensity (*I*), sensory modality (*c*) and power exponent (*a*). *I* is the intensity of the stimulus. In BOA, fitness function is somehow correlated with the intensity of the stimulus *I*. Hence, it can be shown that the more fragrance a butterfly emits (solution quality is better), the easier it is for other butterflies in the environment to sense it and be attracted to it. This relationship is described as follows:

(13) $$f=cI^{a},$$

where *f* denotes fragrance, *c* is the sensory modality, *I* denotes the stimulus intensity, and *a* is the power exponent, which depends on the modality. In this article, we assume values for the parameters *a* and *c* in the range [0,1]. The parameter *a* is a modality-dependent power exponent. It has a variability in absorption and its value may decrease in subsequent iterations. Thus, the parameter *a* can control the behavior of the algorithm, its convergence. The parameter *c* is also important in the perspective of the BOA operation. In theory c∈[0,∞), while in practice it is assumed that c∈[0,1]. The values of *a* and *c* have a significant impact on the speed of the algorithm. Considering this, it should be noted that an important step here is the appropriate selection of these parameters. It should be carried out once for various optimization tasks.

In the BOA we can distinguish the following stages:

- Butterflies in the considered environment emit fragrances that differ in intensity, which results from the quality of the solution. Communication between these animals takes place through sensing the emitted fragrances.
- There are two ways of movement of a butterfly, namely: towards a more intense fragrance emitted by another butterfly and in a random direction.
- Global search is represented by:
  (14) $$x^{new}=x^{old}+\left(r^{2}\;x^{best}-x^{old}\right)f,$$
  where $x^{old}$ is the position of the butterfly (agent) before the move, and $x^{new}$ is the transformation position of the butterfly, $x_{best}$ is the position of the best butterfly in the current population, and *f* is the fragrance of a butterfly $x^{old}$ and *r* denotes a number from the range [0,1] selected in a random way.
- Local search move is formulated by:
  (15) $$x^{new}=x^{old}+\left(r^{2}\;x^{r1}-x^{r2}\right)f,$$
  where $x^{r1}$, $x^{r2}$ are randomly selected butterflies from the population.

At the end of each iteration modifying the population of agents (butterflies), the local search algorithm based on mutation operator (LSAM) is run. This is a significant modification compared to BOA. In this article, the operation of LSAM consisted in the selection of several individuals (solutions) and their transformation with the use of the mutation operator. In case of obtaining better solution after mutation, it replaces the old one. The LSAM algorithm is presented as pseudocode in Algorithm 2. More information regarding the applications of the butterfly algorithm can be found in [31,32,33].
**Algorithm 2** Pseudocode of LSAM operator.1: $x^{r}$—random solution among the top half best agents in population (obtained from BOA).2: $Fit^{r}=F\left(x^{r}\right)$—value of the fitness function for $x^{r}$.3: *I*—number of iterations, ξ—mutation rate.4:                   **Iterative part.**
5: **for**
iterationi=1,2,…,I
**do**6:  Calculate: $\;\;x^{new}=$  Mutate($x^{r},\xi$), $\;\;\;Fit^{new}=Fit\left(x^{new}\right)$.7:  **if** $Fit^{new}<Fit^{r}$ **then**8:    $x^{r}=x^{new}$, $\;\;Fit^{r}=Fit^{new}$.9:  **else**10:    Set a random solution $x^{rnd}$ from the population, but not $x^{r}$.11:    Compute the fitness function $Fit^{rnd}=Fit\left(x^{rnd}\right)$.12:    **if** $Fit^{new}<Fit^{rnd}$ **then**13:     $x^{rnd}=x^{new}$14:    **end if**15:  **end if**16: **end for**

Algorithm 2 includes the process of transforming the individual coordinates of the solution $x=(x_{1},x_{2},\ldots ,x_{n})$ with the use of the mutation operator. The transformation consists in drawing a number from the normal distribution and replacing the old coordinate with a new one. For *j*-th coordinate we use normal distribution:

(16) $$x_{j}^{new}∼N\left(x_{j},\sigma \right),$$

where $x^{j}$ is mean and σ=0.1(uB−lB) is standard deviation. By lB and uB are denoted lower and upper bound of coordinate. Algorithm 3 presents pseudocode of DBOA.
**Algorithm 3** Pseudocode of DBOA.1:                  **Initialization part.**2: Determine parameters of BOA algorithm. *N*—number of butterfly in population, *n*—dimension, *c*—sensor modality and *a*, ξ, *p* parameters.3: Random generate starting population $\left\{x^{1},x^{2},\ldots ,x^{N}\right\}$.4: Calculate the value of the fitness function *F* (hence intensity of the stimulus I=F) for each butterfly $x^{k}\;\left(k=1,2,\ldots ,N\right)$ in population.             **Iterative part.** **for**
iterationi=1,2,…,I
**do**   **for** k=1,2,…,N **do**    Calculate value of fragnance for $x^{k}$ with the use of Equation (13).5:    **end for**    Set the best agent $x_{best}$ among the butterflies.    **for** k=1,2,…,N **do**    Set a random number *r* from range [0,1].     **if** r<p **then**10:      Convert solution $x_{k}^{t}$ in accordance with the Equation (14).     **else**        Convert solution $x_{k}^{t}$ in accordance with the Equation (15).     **end if**   **end for**15:  Change value of the parameter *a*.   Adopt the LSAM algorithm to convert the agents population with mutation rate ξ. **end for** **return**
$x^{best}$.


### 5.3. Aquila Optimizer

Another of the considered algorithms is Aquila Optimizer (AO). This algorithm is a mathematical representation of the hunting behavior of a genus of bird called Aquila (family of hawks). Four main techniques can be distinguished in the way these predators hunt:

- *Expanded exploration*. In the case that a predator is high in the air and wants to hunt other birds, it tilts vertically. After locating the victim from a height, Aquila begins nosediving with increasing speed. We can express this phenomenon with the use of the following equation:
  (17) $$x^{new}=\left(1-\frac{i}{I}\right)\;x^{best}+\left(x^{mean}-rd\;x^{best}\right),$$
  where $x^{new}$ is solution after transformation, $x^{best}$ is the best solution so far and symbolizes position of the prey, *i* is current iteration, *I* is number of maximum iteration and rd is random number from [0,1]. In this case $x^{best}$ can also be defined as the optimization goal or approximate solution. Vector $x^{mean}$ is mean solution from all population:
  (18) $$x^{mean}=\frac{1}{N}\sum_{k=1}^{N}x^{k}.$$
- *Narrowed exploration*. This technique involves circling the prey in flight and preparing to drop the earth and attack the prey. It is also known as short stroke contour flight. This is described in the algorithm by the equation:
  (19) $$x^{new}=Levy_{D}\;x^{best}+x^{random}+rd\;\left(r\cos\phi -r\sin\phi \right),$$
  where $x^{new}$ and $x_{best}$ denotes the same as in expanded exploration point, $x^{random}$ is a random solution from population and rd is a random number from interval [0,1]. Term rcosϕ−rsinϕ simulates spiral flight of Aquila. Expression $Levy_{D}$ is random value of the Levy flight distribution:
  (20) $$Levy_{D}=\frac{s\;u\;\sigma }{{\left|v\right|}^{\frac{1}{\beta }}},$$
  where s,β are constants u,v denote random numbers from range [0,1], and σ is formulated as follows [34]:
  (21) $$\sigma =\frac{\Gamma \left(1+\beta \right)\sin\left(\frac{\pi \beta }{2}\right)}{\Gamma \left(\frac{1+\beta }{2}\right)2^{\frac{\beta -1}{2}}\beta }.$$
  In above equation Γ denotes gamma function. In order to determine the values of the parameters *r* and ϕ the following formula is used:
  (22) $$r=r_{1}+V\;D_{1},\;\;\;\;\theta =-\xi \;D_{1}+\frac{3\pi }{2},$$
  where $r_{1}$ is a fixed integer from {1,2,…,30}, V,ξ are small constants, $D_{1}$ is an integer from {1,2,…,n}.
- *Expanded exploitation*. This hunting technique begins with a vertical attack on a prey, which location is known within some approximation defining the search area. Thanks to this information, Aquila gets as close to its prey as possible. It can be described as follows:
  (23) $$x^{neq}=\alpha \;\left(x^{best}-x^{mean}\right)-rd+\delta \;\left(rd\;(uB-lB)+lB\right),$$
  where $x^{new}$ is the solution after transformation, $x^{best}$ is the best solution at the moment and $x^{mean}$ is the mean solution in all population determined with the use of the formula (18). As before, rd denotes a random number from range [0,1], while lB, uB are lower and upper bound, α and δ are constants parameters of exploitation regulation.
- *Narrowed exploitation*. The characteristic feature of this technique are the stochastic movements of the bird, which attacks the prey in close proximity. It can be described by the formula:
  (24) $$x^{new}=QF\;x^{best}-\left(G_{1}\;rd\;x^{mean}\right)-G_{2}\;Levy_{D}+r_{d}\;G_{1},$$
  where $x^{new}$ denotes solution before transformation, QF is quality function:
  (25) $$QF=i^{\frac{2rd-1}{{\left(1-I\right)}^{2}}}.$$
  $G_{1}$ and $G_{2}$ are described by:
  (26) $$G_{1}=2rd-1,\;\;\;G_{2}=2{\left(t-T\right)}^{2}.$$
  We can adjust the algorithm with the above parameters.

The Aquila’s food-gathering behavior consists of the four hunting techniques previously described. The Formulas (17)–(26) describing four transformations consists in AO algorithm. Algorithm 4 shows description of implementation of the AO algorithm. More about the Aquila Optimizer can be found in [34,35].
**Algorithm 4** Pseudocode of AO.1:                 **Initialization part.**2: Set up parameters of AO algorithm.3: Initialize population in a random way $\left\{x^{1},x^{2},\ldots ,x^{N}\right\}$.4:                    **Iterative part.**5: **for**
iterationi=1,2,…,I
**do**6:  Determine values of the fitness function *F* for each agent in the population.7:  Establish the best solution $x^{best}$ in the population.8:  **for** k=1,2,…,N **do**9:    Calculate mean solution $x^{mean}$ in the population.10:    Improve parameters $G_{1},G_{2},QF$ of the algorithm.11:    **if** $\mathrm{iteration}i\leq \frac{2}{3}I$ **then**12:      **if** rd<0.5 **then**13:        Perform step *expanded exploration* (17) by updating solution $x^{k}$.14:        In the result solution $x^{new,k}$ is obtained.15:        **if** $F\left(x^{new,k}\right)<F\left(x^{k}\right)$ **then** make substitution $x^{k}=x^{new,k}$16:        **end if**17:        **if** $F\left(x^{new,k}\right)<F\left(x^{best}\right)$ **then** make substitution $x^{best}=x^{new,k}$18:        **end if**19:      **else**20:        Perform step *narrowed exploration* (19) by updating solution $x^{k}$.21:        In the result solution $x^{new,k}$ is obtained.22:        **if** $F\left(x^{new,k}\right)<F\left(x^{k}\right)$ **then** make substitution $x^{k}=x^{new,k}$.23:        **end if**24:        **if** $F\left(x^{new,k}\right)<F\left(x^{best}\right)$ **then** make substitution $x^{best}=x^{new,k}$.25:        **end if**26:      **end if**27:    **else**28:      **if** rd<0.5 **then**29:        Perform step *Expanded exploitation* (23) by updating solution $x^{k}$.30:        In the result solution $x^{new,k}$ is obtained.31:        **if** $F\left(x^{new,k}\right)<F\left(x^{k}\right)$ **then** make substitution $x^{k}=x^{new,k}$.32:        **end if**33:        **if** $F\left(x^{new,k}\right)<F\left(x^{best}\right)$ **then** make substitution $x^{best}=x^{new,k}$.34:        **end if**35:      **else**36:        Perform step *narrowed exploitation* (24) by updating solution $x^{k}$.37:        In the result solution $x^{new,k}$ is obtained.38:        **if** $F\left(x^{new,k}\right)<F\left(x^{k}\right)$ **then** make substitution $x^{k}=x^{new,k}$.39:        **end if**40:        **if** $F\left(x^{new,k}\right)<F\left(x^{best}\right)$ **then** make substitution $x^{best}=x^{new,k}$.41:        **end if**42:      **end if**43:    **end if**44:  **end for**45: **end for**46: **return**
$x^{best}$.

## 6. Numerical Example and Test of Algorithms

**Figure 1:**
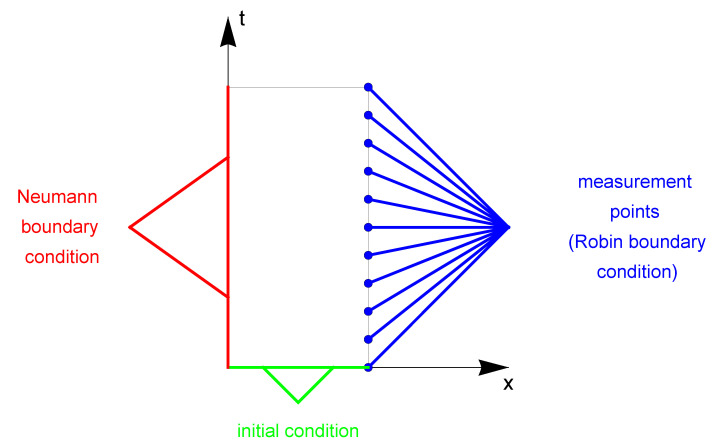
Considered area with marked measuring points (a fragment of the boundary with Neumann boundary condition is marked in red, a fragment of the boundary with Robin boundary condition is marked in blue, and a fragment of the boundary with initial condition is marked in green).

In this section, we present a numerical example illustrating the effectiveness of the algorithms described above. On this basis, the algorithms are compared with each other regarding the inverse problem in the heat flow model. As described in the Section 4, the unknown model parameters that need to be identified are: $\hat{\lambda }$—thermal conductivity, β—order of derivative and *h*—heat transfer function. Temperature measurements on the right boundary of the considered area (Figure 1) are supplementary data necessary to solve the inverse problem. The process should be modeled in a way that allows obtaining temperature values from the mathematical model adjusted to the measurement data. The calculations in the inverse problem are performed on the grid Δx×Δt=100×1995.

In the considered example, the following data are assumed in the model (1)–(4):

$$x_{L}=0,\;x_{R}=3.825,\;t_{end}=71.82,\;c=900,$$

$$\rho =2106,\;T_{\infty }=298,\;\psi \left(x\right)=573.15.$$

The verification of heuristic algorithms is carried out by comparative analysis of the values of the searched parameters obtained from the inverse problem solution with exact values. The exact values of the searched parameters are presented below.

$$\hat{\lambda }=184,\;\beta =1.08,\;h\left(t\right)=2.42t^{2}-5t+78.07.$$

In the case of heat transfer function, the error between the exact function *h*, and the recreated $\hat{h}$ is defined by the following formula:

(27) $$\delta \left(h,\hat{h}\right)=\int_{0}^{t_{end}}\frac{|h\left(t\right)-\hat{h}\left(t\right)|}{\left|h(t)\right|}\;dt.$$

In Table 1 the results obtained for individual algorithms are presented. Evaluating the tested algorithms according to the criterion of the value of the fitness function *F* (9), it is concluded that the DBOA algorithm turned out to be the most appropriate. The value of the fitness function for this algorithm is definitely and significantly lower than in the other cases. Also, the reconstruction errors of the parameters λ and *h* are the smallest for the DBOA algorithm. The second place belongs to the ACO algorithm. Based on the results, it can be seen that minimizing the fitness function is difficult, and the inverse problem is ill-posed. The value of the fitness function (9) is strongly dependent on changes in the values of the searched parameters.

**Figure 2:**
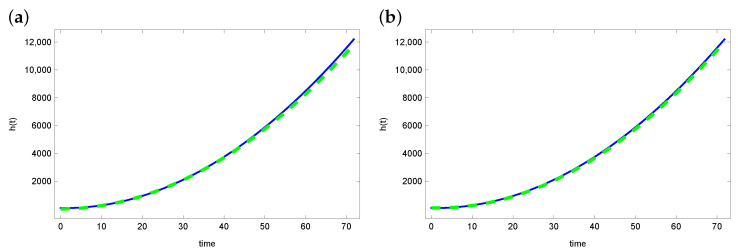
The exact heat transfer h(t) (blue line) and approximate heat transfer (dashed green line) for (**a**) ACO and (**b**) DBOA.

**Figure 3:**
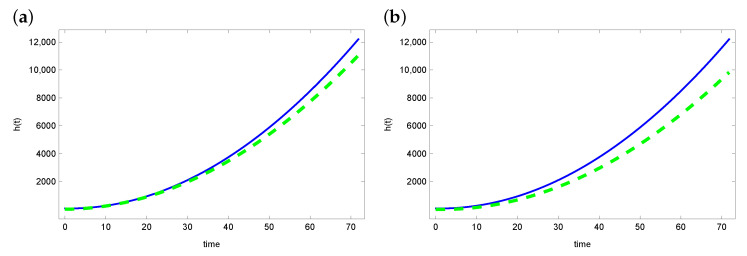
The exact heat transfer h(t) (blue line) and approximate heat transfer (dashed green line) for (**a**) AO and (**b**) BOA.

Figure 2 and Figure 3 present graphs of the exact function *h* and reconstruction function $\hat{h}$ obtained from solving the inverse problem. For the ACO and DBOA algorithms, reconstruction of the function *h* is satisfying. The reconstructed function matches the exact function well. The reconstruction looks a bit worse in the case of the AO and BOA algorithms. Especially in the latter case, the green line (reconstructed *h*) diverges from the blue line (exact function *h*).

We compare the reconstructed temperature values at the measurement points with the measurement data afterwards. The Table 2 presents that the best results are obtained for the DBOA algorithm, and the worst for the BOA algorithm. Generally, these values are not high. Hence, it can be concluded that the reconstructed temperature is well matched to the measurement data, but also that the set problems are ill-posed and difficult to minimize.

**Figure 4:**
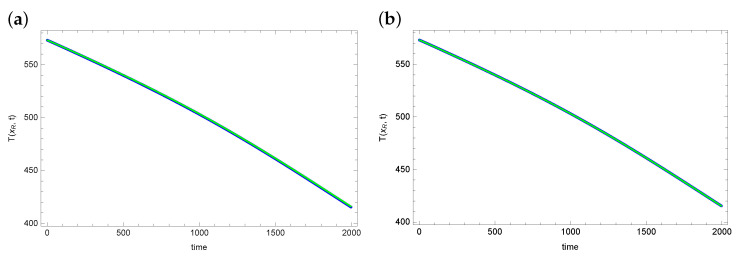
The exact temperature $T(x_{R},t)$ in measurement point (blue line) and reconstructed temperature (green line) for (**a**) ACO and (**b**) DBOA.

**Figure 5:**
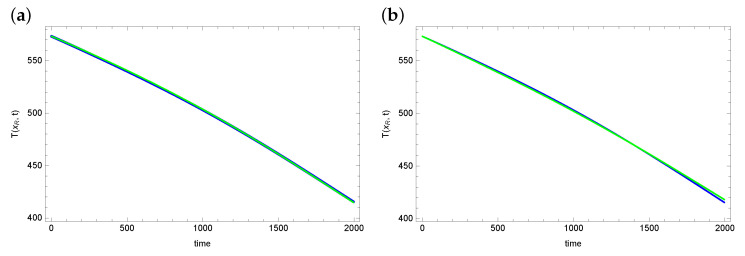
The exact temperature $T(x_{R},t)$ in measurement point (blue line) and reconstructed temperature (green line) for (**a**) AO and (**b**) BOA.

An important parameter evaluating the obtained results is matching the temperature values at the measurement points with the measurement data. Figure 4 and Figure 5 show graphs of reconstructed temperature and graphs of measurement data for each of the algorithms. As can be seen, the reconstructed temperature values are well matched to the measurement data, despite the fact that the reconstructed values of the searched parameters λ and *h* differ significantly for considered algorithms. This proves that the graph of the objective function is flat in the vicinity of the exact solution. Thus, the considered inverse problem is difficult to solve. And the found solution (reconstructed parameter values) may contain significant errors.

## 7. Conclusions

The paper presents the inverse problem of heat flow consisting in the identifying parametric data of the model with given temperature measurements.The unknown parameters of the model are: thermal conductivity, order of fractional derivative and heat transfer function. To solve inverse problem, the function describing the error of the approximate solution should be minimized. Four meta-heuristic algorithms were used and compared, such as: ACO, DBOA, AO and BOA. DBOA turned out to be the best in terms of the value of the minimized function. In the case of DBOA, the value of the minimized function was 0.45, which is a satisfactory result. In the case of other algorithms, these values were much higher: ACO ∼273; BOA ∼2501 and AO ∼482. The DBOA also turned out to be the best in terms of errors in reconstruction model parameters and fitting reconstructed temperature to measurement data. In the case of DBOA, the error of reconstruction the temperature at the measurement points is equal to 0.0131, while for the other algorithms this error was of the order of $10^{-1}$. The considered problems turned out to be difficult to solve. The graph of the fitness function is very flat in the vicinity of the searched solution. Thus, even significant differences in the values of the reconstructed parameters have little impact on the differences in the values of the fitness function.

## Figures and Tables

**Table 1:** Results of calculations ($\bar{\lambda }$—identified value of thermal conductivity coefficient; $\bar{\beta }$—identified value of derivative order; $\bar{h}\left(t\right)$—identified value of heat transfer function; δ—the relative error of reconstruction; *F*—the value of the fitness function).

Algorithm	$\bar{\lambda }$	$\delta_{\bar{\lambda }}\left[\%\right]$	$\bar{\beta }$	$\delta_{\bar{\beta }}\left[\%\right]$	$\bar{h}\left(t\right)$	$\delta_{\bar{h}}$	*F*
ACO	170.86	7.14	1.0838	0.35	$2.27t^{2}+1.41t+10.71$	5.05	272.95
DBOA	178.83	2.81	1.0818	0.17	$2.42t^{2}-7.76t+94.46$	2.39	0.45
AO	124.49	32.34	1.1021	2.04	$2.11t^{2}+1.95t+20.01$	7.76	482.39
BOA	194.27	5.58	1.0798	0.02	$1.98t^{2}-5.42t+7.85$	21.55	2501.21

**Table 2:** Errors of reconstruction temperature function *T* in measurement points ($\Delta_{max}$—maximal absolute error; $\Delta_{mean}$—mean absolute error).

Algorithm	$\Delta_{\max}$	$\Delta_{\mathrm{mean}}$
ACO	0.5111	0.3575
DBOA	0.0261	0.0131
AO	0.7471	0.4361
BOA	2.7409	0.9513

## Data Availability

Not applicable.
